# Trophic Interactions Between Insects and Stream-Associated Amphibians in Steep, Cobble-Bottom Streams of the Pacific Coast of North America

**DOI:** 10.3390/insects3020432

**Published:** 2012-04-10

**Authors:** Trisha Atwood, John S. Richardson

**Affiliations:** Department of Forest Sciences, University of British Columbia, 3041-2424 Main Mall, Vancouver, BC V6T 1Z4, Canada; E-Mail: john.richardson@ubc.ca

**Keywords:** aquatic insects, biofilms, coastal giant salamander, competition, predation, streams, tailed frog, trophic interactions

## Abstract

Two native, stream-associated amphibians are found in coastal streams of the west coast of North America, the tailed frog and the coastal giant salamander, and each interacts with stream insects in contrasting ways. For tailed frogs, their tadpoles are the primary life stage found in steep streams and they consume biofilm from rock surfaces, which can have trophic and non-trophic effects on stream insects. By virtue of their size the tadpoles are relatively insensitive to stream insect larvae, and tadpoles are capable of depleting biofilm levels directly (exploitative competition), and may also “bulldoze” insect larvae from the surfaces of stones (interference competition). Coastal giant salamander larvae, and sometimes adults, are found in small streams where they prey primarily on stream insects, as well as other small prey. This predator-prey interaction with stream insects does not appear to result in differences in the stream invertebrate community between streams with and without salamander larvae. These two examples illustrate the potential for trophic and non-trophic interactions between stream-associated amphibians and stream insects, and also highlights the need for further research in these systems.

## 1. Introduction

Stream foodwebs around the globe are typically dominated numerically and in terms of biomass by invertebrates, particularly the larval stages of aquatic insects. Aquatic insects fill many trophic positions in streams, feeding on algae and biofilms (grazers), plants (herbivores), detritus (detritivores of fine and coarse particles of organic matter), and other animals (predators) [1,2]. Vertebrates living in streams often prey upon stream insects, but also interact in other ways as competitors or through other non-trophic interactions, for instance through bioturbation (disturbing of sediments or the insects themselves). Stream fishes are well known for feeding on invertebrates [3], but larval and adult amphibians in streams can also have impacts on aquatic insect populations [4,5,6]. 

Interactions between amphibians and insects in ponds have demonstrated a variety of competition and predation interactions [7,8,9]. Competition between amphibians and insects has been shown to have strong influences on pond community structure, and occur within both the grazer [9] and predator guilds [10]. Predation interactions between amphibians and insects can go in both directions, with insects preying upon amphibian larvae [11,12], and amphibians, particularly salamanders, feeding on aquatic insects [8,13,14]. Predatory insects (e.g., dytiscids, anisopterans) can influence amphibian communities in ponds at the larval or embryonic stages [15,16]. Alternatively, the effect of aquatic amphibian predators in ponds can be substantial and can influence composition of both pelagic [14] and benthic taxa [8]. To date, most interactions described between amphibians and aquatic insects come from studies conducted in ponds. 

The abundance and diversity of amphibians in stream ecosystems around the planet suggest that they are important ecological components for the assembly of stream insect communities [17,18,19,20], and there is evidence for this from a number of studies, as reviewed by Davic and Welsh [18]. Concern over global declines of amphibian species is exacerbated by the potential for widespread changes to ecosystems and ecosystem services resulting from such species declines [21]. We use two stream-dwelling amphibian species from the Pacific Northwest of North America to illustrate the possible interactions with aquatic insects, and their potential for population- and community-level consequences. These two species are the tailed frog, *Ascaphus truei* Stejneger (Anura: Leiopelmatidae), a species that grazes on rock biofilms as tadpoles, and *Dicamptodon* *tenebrosus* Baird and Girard (Caudata: Dicamptodontidae), the coastal giant salamander, which is predaceous as larvae and adults. We first introduce these species, and then review the evidence for their indirect and direct interactions (competition and predation) with aquatic insects. Finally we explore the potential for higher-order interactions between amphibians and insects in streams [4,5,6,22]. 

### Natural History of *A. truei* and *D. tenebrosus*

The tadpoles of *A. truei* inhabit cold (5–18 °C), perennial streams with steep gradients and coarse substrates within the Pacific Northwest of the United States and southwestern portions of British Columbia, Canada [23]. Coarse (e.g., cobble) substrates provide both relatively large crevices for refuge and flat surfaces for foraging. *Ascaphus truei* are “life-history omnivores”, *i.e.*, as adults they are carnivores feeding on stream and terrestrial insects, however, in their tadpole stage they are herbivores which graze on biofilms on rock surfaces [24]. 

The tadpoles of *A. truei* use their ventrally flattened bodies and a suctorial oral disk with a single toothblade to forage for biofilm on the smooth upper surfaces of cobbles in both riffles and pools within streams [24,25]. They forage primarily at night in an attempt to evade predators that hunt using visual cues, and seek refuge under rocks during the day. Although relatively large in size in comparison to their invertebrate grazer counterparts, *A. truei* tadpoles are quite small compared to other stream vertebrates reaching only ~1 g wet mass before metamorphosis [24]. As a result of their small size, *A. truei* tadpoles fall prey to many aquatic and semi-aquatic predators, e.g., salamanders, frogs, fish, snakes, and shrews [26]. Predators of *A. truei* tadpoles may be an important control on their influence on stream ecosystems, as their predators can impact their grazing by both reducing tadpole abundance through consumption and altering tadpole foraging behavior through trait-mediated effects [26]. Although studies have shown that predators are capable of drastically reducing the abundance of *A. truei* tadpoles [26], they still can represent greater than 90% of the total herbivore biomass in streams they inhabit [25]. Thus, as a result of their superior size compared to many stream macroinvertebrates, *A. truei* tadpoles may be capable of creating competitive pressure among grazers and may have large influences on primary production within their ecosystems [4]. 

Larvae, and occasionally adults, of *D. tenebrosus* inhabit small, low elevation (below 1,200 m) streams along the west coast of North America from north-western California, USA to the south-western portion of British Columbia, Canada [27]. Aquatic stages of *D. tenebrosus* are typically associated with narrow, shaded streams with coarse substrate and an abundance of pools [28,29,30]. Unlike *A. truei*, *D. tenebrosus* do not undergo an ontogenetic shift in trophic position and are predaceous throughout their lives. Giant salamanders can represent over 90% of the predator biomass in small streams [28,31]. 

Aquatic *D. tenebrosus* are sit-and-wait predators, which use chemoreception to detect and locate their prey [32]. *Dicamptodon tenebrosus* feed almost exclusively on stream benthic organisms, although terrestrial insects which have fallen into the stream can make up a substantial portion of their diet [22]. In general, the diet of aquatic *D. tenebrosus* reflects local patterns in prey availability [5,22]. However, despite their generalist predator nature, larger, more active prey taxa appear to be the most frequently consumed by all size classes of aquatic *D. tenebrosus* [22]. Feeding occurs throughout the day and night, although their diel activity patterns suggest that they should be largely feeding at night when they are most active [22]. This type of behavior is hypothesized to occur in response to changes in prey activity and microhabitat use [33]. Due to their large size and generalist feeding behavior, aquatic *D. tenebrosus* act as top predators in many headwater streams.

## 2. Trophic interactions

### 2.1. Amphibians as Competitors with Insects: Competition Between *A. truei* Tadpoles and Grazer Insects

In order to demonstrate the occurrence of competition between two species, a study must first show an adverse effect of one species on the abundance, growth, or survival of another species, and second the study must provide a mechanistic understanding for its results. Studies that successfully incorporate both of these attributes are particularly limited for examining competition between amphibians and stream insects. There are, however, a few studies that provide insight into possible competitive interactions between these two groups, some of which will be described below [4,34,35,36].

Competition between stream insects and amphibians can occur either directly or indirectly. The most common and easily observable competitive interaction is exploitative competition, involving mutual resource depletion by two or more competitors [37]. Another form of competitive interaction between amphibians and stream insects that is often indirectly observed, but rarely directly tested, is interference competition. Interference competition is a direct interaction where one species injures or displaces another species while accessing a shared resource [38]. A third type of competitive interaction called apparent competition, where prey species indirectly depress one another by increasing the abundance of a shared predator [39,40], and might occur between stream insects and amphibians; however, to our knowledge no studies have examined this. Although there are several examples of competition between amphibians and stream insects across all functional feeding groups, we will focus on the competitive interactions between the tadpoles of *A. truei* and stream macroinvertebrates. The relationships between *A. truei* and stream macroinvertebrates are especially interesting because *A. truei* likely exerts both interference and exploitative interactions on stream insects [35]. 

#### 2.1.1. Exploitative Competition

From current research, it is unclear whether *A. truei* tadpoles are effective competitors with grazer insects. However, several characteristics of *A. truei* tadpoles suggest that they have the ability to out compete many grazer insects. First, *A. truei* tadpoles are larger than the majority of stream insects, accounting for 90% of the herbivore biomass in streams they inhabit [25]. Second, *A. truei* tadpoles have much higher metabolisms, requiring them to acquire more food than grazer insects. Third, *A. truei* tadpoles are more mobile than grazer insects and can forage in a larger area. Studies examining exploitative competition between *A. truei* tadpoles and grazer insects, however, have mixed results as to the occurrence of exploitative competition. The first study to investigate exploitative competition between *A. truei* tadpoles and stream macroinvertebrates was conducted by Lamberti *et al.* [4]. This study found that the presence of *A. truei* tadpoles had substantial impacts on periphyton biomass and chlorophyll *a* (Chl *a*) concentrations, reducing them by as much as 98%. In combination with reductions in abundance of periphyton, Lamberti *et al.* [4] found that in some cases *A. truei* tadpoles reduced the abundance of small grazer insects by more than 50%. In contrast to Lamberti *et al.*’s [4] study, Rosenfeld [34] found slight reductions, which were not statistically different, in Chl *a* concentrations and the abundance of grazing chironomids between streams with *A. truei* tadpoles compared to streams without them. Finally, a study by Kiffney and Richardson [25] found that *A. truei* tadpoles did not reduce periphyton biomass. In fact, mean periphyton biomass was slightly, although not significantly, higher than control treatments. With respect to *A. truei’*s effect on grazing insects, the authors found that in the presence of tadpoles, grazing chironomid abundance decreased by 21% and mayfly abundance decreased by as much as 63%. 

The variation in the strength of *A. truei* tadpole interactions with grazer insects may be due to differences in environmental factors (both biotic and abiotic). Several studies have documented that interaction strengths are variable with time [41,42,43] and that even small changes in environmental factors (e.g., temperature) can influence the strength of species interactions [44]. Environmental changes can influence species interactions by affecting the performance of one or multiple species, thus influencing how that species interacts with other species. Several factors have been found to influence *A. truei*’s interactions with both other grazers and their food resource. For example, one study found that *A. truei* had a greater effect on grazer mayfly abundance in nutrient enriched treatments than non-enriched treatments [35]. Another study found that periphyton in some British Columbian streams was controlled by *A. truei* grazing, while in other streams periphyton was controlled by light levels despite the presence of *A. truei* tadpoles [36]. The variation in the results presented above suggests that the outcomes of interactions between *A. truei* tadpoles and grazer insects are complex and perhaps context dependent. In most cases the studies presented above suggest a combination of exploitative and interference competition. 

#### 2.1.2. Interference Competition

Consumer traits have strong influences on community interactions [38,45,46] and body size has been used to identify “key” or “dominant” species within a system [47]. In general, *A. truei* tadpoles are larger than most common macroinvertebrate grazers. Upon hatching, *A. truei* tadpoles are ~11 mm in length and grow to a maximum of ~65 mm close to the time of metamorphosis [48]. The final length of *A. truei* tadpoles is over twice that of the trichopteran *Dicosmoecus gilvipes* (Hagan), a large grazer insect common in streams of the Pacific Northwest, USA [49]. *Ascaphus truei*’s larger size almost inevitably conveys an advantage for space over macroinvertebrate grazers. However, antagonistic encounters, and thus the potential for interference competition, have only been indirectly observed [35,50]. 

A study done by Kiffney and Richardson [35] found evidence that suggests interference competition may be occurring between *A. truei* and stream insect grazers. In this study, the authors examined the individual and joint effects of *A. truei* tadpoles and nutrient additions on periphyton biomass and insect grazer abundance in experimental stream channels. This study showed that the presence of *A. truei* tadpoles decreased insect grazer abundance. However, tadpoles were not able to significantly reduce periphyton biomass. Additionally, despite a substantial increase in the biomass of their shared periphyton resource, grazer insect abundance in the presence of tadpoles was in some cases lower than control treatments. Results from this study show complex interactions; however, they do suggest the possibility of antagonistic behavior by *A.truei* towards insect grazers. 

#### 2.1.3. Difference in Resource Use

The level of niche and functional overlap between *A. truei* tadpoles and stream insects is important for understanding how stream ecosystem function will be impacted should *A. truei* tadpole populations decline due to anthropogenic disturbances, such as forest harvest. However, the details of differences in resource use between *A. truei* tadpoles and stream insects are unknown. The mouthparts of *A. truei* and most insect grazers differ in terms of selectivity and how closely they can crop biofilms from stone surfaces [45]. With increased densities of *A. truei* tadpoles there was a decelerating rate of loss of algae [36], suggesting algae may have a spatial refuge from *A. truei* if they are very small or in crevices of rocks. By virtue of their smaller body size and smaller mandibles, grazer insects (e.g., *Glossosoma* and *Stenonema*) may be able to further reduce biofilm standing crop beyond what *A. truei* is capable of [45], but this hypothesis requires testing. There are additional questions regarding resource use and potential interactions between *A. truei* tadpoles and grazer insects that warrant further investigation. (1) To what extent do dietary components overlap between *A. truei* tadpoles and grazer insects? (2) What is the extent of spatial overlap between species? (3) What is the extent of temporal (diurnal and seasonal) overlap between species? (4) What is the total overlap among species with respect to space, time, and diet dimensions? (5) Which species of grazer insects are the most similar (or dissimilar) to *A. truei* tadpoles with respect to niche? (6) Which grazer insect species pose the greatest competitive threat to *A. truei* tadpoles?

### 2.2. Predation

Aquatic forms of *D. tenebrosus* are voracious and abundant predators in streams they inhabit [22]. Murphy and Hall [31] reported that larval *D. tenebrosus* represented 90% of the predator biomass in small Oregon and northern Californian streams. Larval *D. tenebrosus* are obligate benthic predators, feeding on stream and some drowned terrestrial macroinvertebrates and tadpoles [22]. Although larval *D. tenebrosus* are more active at night, Parker [22] found no difference in stomach content mass and the proportion of intact prey between night and day, suggesting that larval *D. tenebrosus* are opportunistic predators, feeding whenever prey presents itself. Due to their opportunistic feeding behavior and their large abundance, especially in fishless streams, aquatic *D. tenebrosus* may have strong influences on stream macroinvertebrate abundance and exert indirect effects on algal and detrital biomass and assemblages through tri-trophic cascades. 

Although larval and neonate *D. tenebrosus* act as top aquatic predators in fishless streams, intraguild predation can occur in streams containing salmonids [5,51]. Adult and older *Dicamptodon* larvae can defend themselves from fish and other conspecific predators both behaviorally and with noxious skin secretions, however, young-of-year *Dicamptodon* larvae lack chemical defenses [51]. As a result, young-of-the-year *Dicamptodon* need adequate refuge from predators, which may also explain the correlation between the presence of larval *D. tenebrosus* and coarse benthic substrates. Although fish can effectively prey on *Dicamptodon* salamanders, they also act as competitors for food resources, as benthic macroinvertebrates make up a sizeable portion of stream salmonid [52] and larval *D. tenebrosus* diets [22]. Thus, predators of *D. tenebrosus* may influence the impacts of this species on stream communities both through consumption and exploitative competition.

There have been few considerations of the impacts of *D. tenebrosus* as top predators in stream communities. However, in one such experiment three pools in a stream were divided medially with mesh dividers to create three pairs of enclosures, with one of each pair having giant salamander larvae at natural densities, and the other member of each pair with all salamanders removed [53]. The presence of *D. tenebrosus* resulted in large reductions in the densities of large-bodied invertebrate species, of which many were predaceous aquatic insects. However, some smaller-bodied insects, such as *Baetis* spp. and orthocladiine chironomids, actually increased in the presence of the salamander larvae, suggesting an indirect effect of the salamanders by releasing these small species from predation pressure from the larger invertebrates [53]. 

To examine whether giant salamander larvae have a detectable effect on benthic communities in another set of small streams, we collected five benthic samples from each of 18 streams in the Chilliwack area of southwestern British Columbia [54]. We contrasted the stream insect communities of streams with and without (*i.e.*, not detected) giant salamander larvae, with nine of each category of stream. Canonical discriminant analysis was used to test for differences associated with salamander presence in the relative abundances of benthic invertebrates (PROC CANDISC, SAS ver. 9.2, SAS Inc, Cary, NC). There was no significant difference (*p* = 0.44, n = 18) found between streams with or without salamanders. This suggests that while these salamanders are the top predator in many streams, they do not always lead to shifts in community structure, and that perhaps their effects are context-dependent. 

## 3. Population and Community-Level Consequences of Amphibian-Insect Trophic Interactions

Stream insects and amphibians interact through trophic and non-trophic mechanisms. However, there has been very little consideration of the population dynamical effects of these interactions, despite evidence they may be considerable. Moreover, there may be community-scale consequences of these interactions. 

Interactions between pond amphibians and insects have been the subject of many experiments. In pond mesocosms, the competitive effects of aquatic insects on growth of *Hyla* and *Bufo* tadpoles was of a similar or greater magnitude to that of the two amphibian species on each other [7]. The mechanism for that competitive effect was demonstrated to be depletion of the periphyton resource consumed by both of the amphibian tadpoles and the aquatic insects [7]. Study of tailed frog tadpoles at a range of densities has demonstrated the ability of that species to depress periphyton resources in streams [36], but as with other studies, the consequences for insect populations are rarely determined. 

## 4. Conclusions

Above we have reviewed the limited evidence of how two particular species of stream amphibians might interact with aquatic insects. Many amphibians have complex life cycles in which different life stages may occupy different trophic levels. We have used these species as examples of the potential for interesting competitive and predator-prey interactions that might have large roles in stream foodwebs. Given the conservation concerns for amphibian populations globally, the reduction or local losses of stream amphibians may have profound effects for stream communities and ecosystem functions [18]. A greater appreciation for the roles of amphibians in stream communities would further support efforts being made to conserve species and their habitats. 

An emphasis in the literature for stream insects has been on the effects of predaceous fish on the daily timing of foraging behavior of insects, and to some extent the population dynamical consequences of fish predation [7,8,9]. There have been few studies that have detailed the interactions and quantitative effects of amphibians on stream insects [4,5,35]. Given that amphibian biomass can be considerable in some streams, and in some cases fill the role of top predator, it is surprising that there has been so little consideration of the population-level interactions between stream insects and amphibians. There is great opportunity to explore the impacts of amphibians on stream insect populations given that the densities of these amphibians are easily manipulated experimentally. 
